# Association Between Pneumococcal Vaccination Uptake and Loneliness Among Regular Patients in Rural Community Hospitals: A Cross-Sectional Study

**DOI:** 10.7759/cureus.65293

**Published:** 2024-07-24

**Authors:** Ryuichi Ohta, Toshihiro Yakabe, Hiroshi Adachi, Chiaki Sano

**Affiliations:** 1 Community Care, Unnan City Hospital, Unnan, JPN; 2 Family Medicine, Unnan City Hospital, Unnan, JPN; 3 Community Medicine Management, Shimane University Faculty of Medicine, Izumo, JPN

**Keywords:** pneumococcal infections, pneumococcal infection prevention & control, psychosocial factors, cross-sectional studies, rural population, loneliness, vaccination

## Abstract

Introduction

Vaccination is essential for preventing infectious diseases such as pneumonia and seasonal viral infections. The COVID-19 pandemic has underscored the critical role of vaccination in public health. However, vaccination uptake can be influenced by biopsychosocial conditions. Immunocompromised individuals, for instance, face restrictions with live vaccines, and psychosocial factors like loneliness can negatively impact attitudes towards vaccination. This study aims to clarify the association between loneliness and pneumococcal vaccination rate among regular patients in a rural Japanese community.

Method

A cross-sectional study was conducted at Unnan City Hospital in Unnan City, a rural area in southeastern Shimane Prefecture, Japan. Participants included patients over 40 who regularly visited the general medicine department between September 1, 2023, and November 31, 2023. Data on vaccination rates for pneumococcal pneumonia and loneliness levels assessed using the Japanese version of the three-item University of California, Los Angeles (UCLA) Loneliness Scale were collected. Additional data on demographics, BMI, renal function, and comorbidities were extracted from electronic medical records. Statistical analyses were performed to identify factors associated with vaccination rates, including univariate and multivariate logistic regression.

Results

Out of 1,024 eligible patients, 647 participated in the study. Participants with higher loneliness had significantly lower vaccination rates for pneumococcal pneumonia (22.3% vs. 34.2%, p = 0.001). The multivariate logistic regression model showed that higher loneliness was significantly associated with lower vaccination likelihood (odds ratio (OR) = 0.54, 95% CI = 0.37-0.78, p = 0.0011). Age was positively associated with vaccination (OR = 1.08, 95% CI = 1.06-1.11, p < 0.001), whereas higher comorbidity scores (Charlson Comorbidity Index (CCI) ≥ 5) and frequent healthy eating practices were associated with lower vaccination rates.

Conclusion

This study demonstrates a significant association between higher loneliness levels and lower pneumococcal vaccination rates among patients in a rural Japanese community. Addressing psychosocial barriers such as loneliness could enhance vaccination uptake. Public health interventions focused on reducing loneliness and enhancing social support are essential to improving vaccination rates and preventing infectious diseases. Further research should explore the causal mechanisms and develop targeted strategies to mitigate the impact of loneliness on health behaviors.

## Introduction

Vaccination prevents infectious diseases like pneumonia and seasonal viral infections [1]. The global COVID-19 pandemic has significantly underscored the importance of vaccination, bringing widespread recognition to its critical role in public health [2,3]. By mitigating the risk of vaccine-preventable diseases, vaccinations are a fundamental component in reducing the burden of infectious diseases and improving overall health outcomes [4]. Consequently, promoting vaccinations is crucial to minimizing health complications and ensuring better health conditions for populations worldwide [5].

Individuals' biopsychosocial conditions can significantly influence the motivation to receive vaccines. For instance, immunocompromised individuals may face restrictions in receiving live vaccines due to the heightened risk of potential infections [6,7]. In such cases, medical professionals are vital in facilitating appropriate vaccination strategies, ensuring these individuals receive the necessary protection without compromising their health [8]. Additionally, psychosocial factors profoundly impact attitudes toward vaccination. Exposure to negative information regarding vaccines can shape people's perceptions and attitudes, often fostering fear and depressive feelings [9]. This fear can be exacerbated by a lack of social interaction, leading to feelings of loneliness and further discouraging individuals from seeking vaccination [10].

Addressing these psychosocial barriers is critical to improving vaccination rates. By mitigating negative attitudes toward vaccines and addressing the isolation and loneliness that may accompany such attitudes, we can enhance the overall acceptance and uptake of vaccinations [11,12]. While the relationship between loneliness and vaccination rates has not been extensively studied, improving social interactions and reducing feelings of loneliness could positively influence individuals' willingness to vaccinate.

This research aims to clarify the association between pneumococcal vaccination rates and loneliness. In Japan, the yearly incidence rate of pneumococcal infections among older people is approximately 200 cases per 100,000 person-years for pneumococcal pneumonia and 10-20 cases per 100,000 for invasive pneumococcal disease, impinging on older people's lives [11,12]. Understanding this relationship is essential for developing targeted interventions to improve pneumococcal vaccination uptake, particularly among individuals experiencing social isolation. By exploring the biopsychosocial factors influencing vaccination motivation, this study seeks to contribute valuable insights into public health strategies that promote higher pneumococcal vaccination rates and, ultimately, better community health outcomes.

## Materials and methods

This cross-sectional study involved rural residents who were frequent visitors to a community hospital in a rural area of Japan. The aim was to investigate the relationship between the vaccination rate for pneumococcal pneumonia and the levels of loneliness experienced by these regular patients.

Setting

Unnan City, situated in the southeastern part of Shimane Prefecture, is among the most rural areas in Japan. As of 2020, the city's population totaled 37,638, with 18,145 men and 19,492 women. Notably, 39% of the population was over 65, and this figure is projected to rise to 50% by 2025. Unnan City is served by 16 clinics, 12 home care stations, three visiting nurse stations, and a single public hospital, Unnan City Hospital. At the time of this study, Unnan City Hospital had 281 beds, categorized into 160 acute care beds, 43 comprehensive care beds, 30 rehabilitation beds, and 48 chronic care beds [13]. Care managers, working independently or within home care stations, collaborate with home care patients, their families, and other medical and care professionals. They oversee the patients' care plans and assess the necessity for additional professional services. Home care workers affiliated with these stations provide physical care, assisted living support, and transportation services to enhance the quality of life for home care patients [14].

Participants

The study included all patients over 40 who regularly visited the Department of General Medicine at Unnan City Hospital between September 1, 2023, and November 31, 2023 [15]. Data were collected by extracting information from the electronic medical records of patients who regularly attended the hospital for chronic disease management or annual health check-ups.

Data collection

Primary Outcome

The study's primary outcome is the rate of vaccination for pneumococcal pneumonia. Although this vaccine is not mandatory in Japan, increasing the vaccination rate can significantly reduce the incidence of pneumococcal pneumonia, improving overall health conditions. Participants were asked the following question in the questionnaire: "Have you received a pneumococcal pneumonia vaccine within the last five years?"

Independent Variables

The degree of loneliness was assessed using the Japanese version of the three-item University of California, Los Angeles (UCLA) Loneliness Scale among community-dwelling adults (score range: 3-9). The scale includes three items: Item 1, "How often do you feel you lack companionship?" (scale of one to three); Item 2, "How often do you feel left out?" (scale of one to three); and Item 3, "How often do you feel isolated from others?" (scale of one to three). The total score was calculated by summing the scores for each item [16].

Covariates

Regarding exercise, the participants were asked as follows: “Do you engage in regular exercise? (For this question, "exercise" refers to physical activity performed at least three times a week, sustained for a minimum of 10 minutes per session.)” The participants answered the question with a yes or no. 

Regarding eating habits, the participants were asked as follows: “How many days per week do you have at least two meals consisting of three components: a staple food (such as rice, bread, or noodles), a main dish (using meat, fish, eggs, or soy products), and a side dish (prepared with vegetables, mushrooms, tubers, or seaweed)?” The participants answered the question less than once, two to three times weekly, four to five times weekly, and more than five times weekly [12,17]. 

Regarding sleep, the participants were asked as follows: “Do you make an effort to get sufficient sleep? (In this context, "sufficient sleep" is defined as a minimum of six hours per night.)” The participants answered the question with a yes or no.

Background data of the participants were collected from the electronic patient records of Unnan City Hospital [15]. The collected data included age, sex, body mass index for nutritional assessment, serum creatinine level (mg/dL), estimated glomerular filtration rate (eGFR) (mL/min/1.73 m²) for renal function assessment, and the Charlson Comorbidity Index (CCI) for evaluating the severity of comorbidities such as heart failure, myocardial infarction, asthma, chronic obstructive pulmonary disease, kidney disease, liver disease, diabetes mellitus, brain infarction, brain hemorrhage, hemiplegia, connective tissue diseases, dementia, and cancer [17]. The laboratory data were taken from the participants' most recent visits to the hospital for their chronic diseases or annual health checks [15].

Statistical analysis

Student’s t-test was employed to analyze parametric data, while the Mann-Whitney U test was used for nonparametric data. The loneliness score was dichotomized into two groups: ≥4 (higher loneliness) and <4 (lower loneliness), given that the mean and median were similar (mean: 4.17; standard deviation (SD): 1.42; median: 4; interquartile range: 2). The following variables were dichotomized: agricultural activities, not frequent (less than once weekly, two to three times weekly) and frequent (four to five times weekly, and more than five times weekly); eating habits, less frequent (less than once weekly, two to three times weekly) or frequent (four to five times weekly and more than five times weekly); and CCI, over five or less than five. A univariate regression model was utilized to determine if the vaccination rate was associated with independent variables. Multivariate logistic regression analysis was then conducted to explore the relationship between vaccination rate and higher loneliness. Independent variables that showed a correlation with agricultural activities in the univariate regression analysis (p-value < 0.1) and those used in previous studies were included in the multivariate logistic model [15]. Participants with missing data were excluded from the analysis. Statistical significance was defined as p < 0.05. All statistical analyses were performed using EZR software (Saitama Medical Center, Jichi Medical University, Saitama, Japan), which serves as a graphical user interface for the R software (The R Foundation, Vienna, Austria) [18].

Ethical considerations

The hospital was assured of the anonymity and confidentiality of the patient information used in this study. Information related to this study was posted on the hospital website without disclosing any patient details. The contact information of the hospital representative was also listed on the website to ensure that any questions regarding this study were addressed. All participants were informed of the purpose of this study and provided informed consent. The Unnan City Hospital clinical ethics committee approved the study protocol (approval code: 20230010).

## Results

Participant selection

Between September 1, 2023, and November 31, 2023, 1,024 patients were regularly followed by the general medicine department. The questionnaires were sent to all the patients. In total, 647 participants who answered the questionnaires were included in this study [15]. 

Demographics of the participants

The mean age of the participants was 71.26 years (SD = 12.18), with a slightly higher representation of females (53.7%). The BMI of participants with higher loneliness was significantly lower (22.62 kg/m^2^) compared to those with lower loneliness (23.41 kg/m^2^) (p = 0.009). Additionally, those with higher loneliness also had lower weights (56.99 kg) compared to those with lower loneliness (59.68 kg) (p = 0.007). Participants who frequently engaged in healthy eating practices were significantly fewer in the higher loneliness group (18.4%) compared to the low loneliness group (25.2%) (p = 0.037). Moreover, good sleep was more prevalent among those with lower loneliness (85.8%) compared to those with higher loneliness (80.7%), though this difference was not statistically significant (p = 0.093). The rate of pneumococcal vaccination was significantly lower in the higher loneliness group (22.3%) compared to the lower loneliness group (34.2%) (p = 0.001). Participants with higher loneliness had significantly higher mean scores in the UCLA Loneliness Scale items. The scores for companionship (mean = 2.04, SD = 0.49), feeling isolated (mean = 1.57, SD = 0.58), and feeling left out (mean = 1.63, SD = 0.57) were all significantly higher in the higher loneliness group compared to the lower loneliness group, where all items had a mean score of 1.00 (p < 0.001 for all items). The CCI distribution did not show significant differences between the two groups regarding most individual CCI scores. However, a CCI score of five or more was more common among those with lower loneliness (36.1%) compared to those with higher loneliness (31.5%), though this difference was not statistically significant (p = 0.213) (Table 1).

**Table 1:** The demographics of the participants based on the amount of loneliness BMI: body mass index; CCI: Charlson Comorbidity Index; eGFR: estimated glomerular filtration rate; SD: standard deviation. The loneliness score was dichotomized into two groups: ≥4 (higher loneliness) and <4 (lower loneliness), given that the mean and median were similar (mean: 4.17; standard deviation (SD): 1.42; median: 4; interquartile range: 2). Companionship, "How often do you feel you lack companionship?" (scale of one to three); Leftover, "How often do you feel left out?" (scale of one to three); Isolated, "How often do you feel isolated from others?" (scale of one to three).

Factor	Total	Low loneliness	Higher loneliness	p-value
N	647	146	501	
Age, year old, mean (SD)	71.26 (12.18)	71.85 (11.37)	70.71 (12.87)	0.232
Male sex, n (%)	299 (46.3)	148 (47.9)	151 (44.8)	0.477
BMI, mean (SD) (kg/m^2^)	23.00 (3.81)	23.41 (3.47)	22.62 (4.07)	0.009
Height, cm, mean (SD)	158.63 (8.60)	159.08 (8.70)	158.21 (8.51)	0.196
Weight, kilogram, mean (SD)	58.28 (12.79)	59.68 (12.35)	56.99 (13.07)	0.007
eGFR, mL/min/1.73m^2^, mean (SD)	0.63 (0.48)	0.64 (0.48)	0.63 (0.48)	0.805
Albumin, g/dL, mean (SD)	4.10 (0.41)	4.09 (0.41)	4.10 (0.41)	0.946
Frequent healthy eating, n (%)	142 (21.9)	57 (18.4)	85 (25.2)	0.037
Exercise, n (%)	267 (41.3)	126 (40.6)	141 (41.8)	0.811
Good sleep, n (%)	538 (83.2)	266 (85.8)	272 (80.7)	0.093
Pneumonia vaccination, n (%)	181 (28.0)	106 (34.2)	75 (22.3)	0.001
Companionship, mean (SD)	1.54 (0.63)	1.00 (0.00)	2.04 (0.49)	<0.001
Isolated, mean (SD)	1.30 (0.51)	1.00 (0.00)	1.57 (0.58)	<0.001
Leftover, mean (SD)	1.33 (0.52)	1.00 (0.00)	1.63 (0.57)	<0.001
CCI ≥ 5, n (%)	218 (33.7)	112 (36.1)	106 (31.5)	0.213
CCI, n (%)				
0	20 (3.1)	7 (2.3)	13 (3.9)	
1	57 (8.8)	25 (8.1)	32 (9.5)	
2	82 (12.7)	38 (12.3)	44 (13.1)	
3	142 (21.9)	72 (23.2)	70 (20.8)	
4	128 (19.8)	56 (18.1)	72 (21.4)	
5	107 (16.5)	58 (18.7)	49 (14.5)	
6	64 (9.9)	34 (11.0)	30 (8.9)	
7	34 (5.3)	17 (5.5)	17 (5.0)	
8	10 (1.5)	2 (0.6)	8 (2.4)	
9	3 (0.5)	1 (0.3)	2 (0.6)	

Results of the logistic regression analysis

A multivariate logistic regression model was employed to determine the factors associated with pneumococcal vaccination rates. Higher loneliness was associated with a significantly lower likelihood of receiving the pneumococcal vaccination (odds ratio (OR) = 0.54, 95% CI = 0.37-0.78, p = 0.0011). Other significant factors included age, with older participants being more likely to be vaccinated (OR = 1.08, 95% CI = 1.06-1.11, p < 0.001) and having a CCI score of five or more, which was associated with a lower likelihood of vaccination (OR = 0.50, 95% CI = 0.31-0.79, p = 0.0033). Participants who ate healthily frequently were less likely to be vaccinated (OR = 0.59, 95% CI = 0.36-0.97, p = 0.038) (Table 2).

**Table 2:** The multivariate logistic regression model with vaccination of pneumococcal pneumonia, loneliness, and health-related factors BMI: body mass index; CCI: Charlson Comorbidity Index; CI: confidential interval. The loneliness score was dichotomized into two groups: ≥4 (higher loneliness) and <4 (lower loneliness), given that the mean and median were similar (mean: 4.17; standard deviation (SD): 1.42; median: 4; interquartile range: 2).

Factor	Odds ratio	95%CI	p-value
Higher loneliness	0.54	0.37-0.78	0.0011
Age	1.08	1.06-1.11	<0.001
BMI	1.00	0.95-1.06	0.98
CCI ≧5	0.50	0.31-0.79	0.0033
Healthy eating	0.59	0.36-0.97	0.038
Adequate sleep	1.15	0.67-1.96	0.62
Regular exercise	0.97	0.67-1.41	0.86
Albumin	1.34	0.79-2.25	0.28

## Discussion

The present study highlights a significant association between higher loneliness and lower vaccination rates for pneumococcal pneumonia among patients in a rural Japanese community. This finding underscores the critical role of psychosocial factors in health behaviors, particularly vaccination uptake. The results align with previous research indicating that social isolation and loneliness can adversely affect health outcomes and health-related behaviors [19,20]. Our study contributes to the growing body of literature emphasizing the importance of addressing psychosocial barriers to improve vaccination rates and public health outcomes.

One of the key findings of this study is that individuals experiencing higher levels of loneliness were less likely to receive pneumococcal vaccinations. This association persisted even after adjusting for various confounding factors, including age, BMI, and comorbidities. These results suggest that loneliness might be a barrier to healthcare services, including vaccinations. Similar observations have been made in other contexts where social support and community engagement positively influence health behaviors and preventive care utilization [21,22]. Patients with higher loneliness may have various anxieties associated with their health conditions. They may also be isolated from their communities and lack medical information regarding health prevention, including vaccinations [23]. To promote their health conditions, isolated people with higher loneliness should be supported by communities and informed effectively regarding vaccination from medical institutions and local governments.

The study also found that older participants were more likely to be vaccinated, which could be attributed to increased awareness of the risks associated with pneumococcal infections in older adults. Conversely, participants with higher comorbidity scores (CCI≥5) were less likely to be vaccinated, highlighting the potential challenges faced by individuals with multiple health issues in accessing preventive services. This finding is consistent with prior research identifying multimorbidity as a significant barrier to preventive healthcare uptake [24]. Patients with multimorbidity can have critical pneumonia because of immunocompromised conditions affected by chronic diseases such as hypertension, diabetes, heart failure, and chronic lung diseases [25,26]. Pneumococcal pneumonia is one of the critical diseases for older and immunocompromised patients. Vaccinations for pneumococcal pneumonia can prevent the infection and its severity [1]. Focusing on older patients with multimorbidity should be emphasized to improve community health in aging societies.

Interestingly, participants who frequently engaged in healthy eating practices were less likely to be vaccinated. This counterintuitive result may reflect a complex interplay of health beliefs and behaviors [27,28]. Individuals who prioritize healthy eating might perceive themselves as less vulnerable to infections, reducing their perceived need for vaccinations [29]. On the other hand, they may not know the concrete effectiveness of vaccination regarding pneumococcal infections [30]. The evidence about the vaccination of pneumococcal pneumonia shows that the vaccination can prevent critical situations of older patients' pneumonia [1]. Further research is needed to explore the underlying reasons for this association and the effects of appropriate information provisions on the change in vaccination uptake.

This study has several limitations that need to be acknowledged. Firstly, the cross-sectional design precludes the establishment of causal relationships between loneliness and vaccination rates. Longitudinal studies are needed to determine the directionality of this association. Secondly, the study was conducted in a single rural community in Japan, which may limit the generalizability of the findings to other populations and settings. Rural communities may have unique social and healthcare dynamics that differ from urban areas.

Additionally, relying on self-reported measures for vaccination status and loneliness may introduce recall or social desirability bias. Future studies could benefit from using objective measures of vaccination uptake and more comprehensive assessments of social isolation and loneliness. Lastly, the study did not account for other potential confounders, such as socioeconomic status, access to healthcare, and cultural factors that might influence vaccination behaviors.

## Conclusions

This study demonstrates a significant association between higher levels of loneliness and lower rates of pneumococcal vaccination among patients in a rural Japanese community. These findings highlight the importance of addressing psychosocial factors, particularly loneliness, to improve vaccination uptake and overall health outcomes. Public health interventions to reduce loneliness and enhance social support could be crucial in promoting vaccination and preventing infectious diseases. Future research should focus on exploring the causal mechanisms underlying this association and developing targeted strategies to mitigate the impact of loneliness on health behaviors. By incorporating these findings into public health strategies, healthcare providers and policymakers can better address the barriers to vaccination, ultimately contributing to improved health outcomes and a reduced burden of infectious diseases in communities.
